# Prevalence and Potential Risk Factors of *Helicobacter pylori *Infection among Asymptomatic Individuals in Kazakhstan

**DOI:** 10.31557/APJCP.2021.22.2.597

**Published:** 2021-02

**Authors:** Linda Mezmale, Inese Polaka, Dace Rudzite, Reinis Vangravs, Ilze Kikuste, Sergei Parshutin, Ilva Daugule, Altynbek Tazhedinov, Tatyana Belikhina, Nurbek Igissinov, Jin Young Park, Rolando Herrero, Marcis Leja

**Affiliations:** 1 *Institute of Clinical and Preventive Medicine, University of Latvia, Riga, Latvia. *; 2 *Faculty of Medicine, University of Latvia, Riga, Latvia. *; 3 *Riga East University Hospital, Riga, Latvia. *; 4 *Digestive Disease Centre GASTRO, Riga, Latvia. *; 5 *Regional Diagnostic Centre, Almaty, Kazakhstan.*; 6 ^*6*^ *Semey Regional Oncology Centre, Semey, Kazakhstan. *; 7 *Astana Medical University, Nur-Sultan, Kazakhstan. *; 8 *Central Asian Cancer Institute, Nur-Sultan, Kazakhstan. *; 9 *Eurasian Institute For Cancer Research, Bishkek, Kyrgyzstan. *; 10 *Prevention and Implementation Group, Section of Early Detection and Prevention, International Agency for Research on Cancer, Lyon, France. *; 11 *Agencia Costarricense de Investigaciones Biomédicas, San José, Costa Rica. *

**Keywords:** Helicobacter pylori, risk factors, prevalence, Kazakhstan, gastric cancer

## Abstract

**Background:**

*Helicobacter pylori* (*H. pylori*) infection is associated with several risk factors such as demographic, socioeconomic status and personal habits, which vary in different populations. This is the most up-to-date data on *H. pylori* prevalence and potential risk factors for *H. pylori* infection among asymptomatic middle-aged individuals in Kazakhstan.

**Methods:**

Apparently healthy individuals aged 40 to 64, who took part in the health control in the outpatient clinic, were invited to participate in the study; answered a questionnaire, donated a blood sample. The antibodies to *H. pylori* were analysed by latex agglutination method. The baseline characteristics of study subjects with or without *H. pylori* infection were compared using the Chi-square test. Odds ratio (OR) and 95% confidence intervals (CI) for the association between *H. pylori* infection and potential risk factors were estimated using multivariable logistic regression models.

**Results:**

Altogether 166 subjects (59% male; the median age - 51 years old) were included; 104 (62.7%) were *H. pylori* positive. There were no statistically significant differences between *H. pylori* positive and *H. pylori* negative groups in respect to the gender, anthropometric measurements, socioeconomic factors and personal habits. The multiple variable analysis showed that age (OR, 1.99; 95% CI, 1.03 – 3.86; P=0.04) and increased salt intake (OR, 2.21; 95% CI, 1.12 – 4.35; P=0.02) were associated with *H. pylori* infection.

**Conclusions:**

More than half of the study subjects were infected with *H. pylori* in Kazakhstan. The prevalence of *H. pylori* infection was independently associated with older age and regular high salt consumption.

## Introduction

*Helicobacter pylori* (*H. pylori*) infection is still a significant public health problem worldwide (Hooi et al., 2017), and it is the major risk factor for the development of gastric cancer (Pereira-Marques et al., 2019; Wroblewski et al., 2010). Despite a decreasing trend in incidence rates during recent decades, it is still among the most common cancers and leading causes of cancer death globally (Igissinov et al., 2018). De Martel et al., (2020) reported that 810,000 new cases of gastric cancer (mainly non-cardia gastric adenocarcinoma) were attributed to chronic *H. pylori* infection in 2018. In Kazakhstan, gastric cancer ranks fourth in incidence among all types of cancer (Globocan., 2018). However, the incidence of gastric cancer tends to decrease, which is associated with successful prevention of this pathology (Igissinov et al., 2018). The effective means of preventing gastric cancer in the developed world include diet, lifestyle modification, as well as successful *H. pylori* testing and treatment (Rawla and Barsouk, 2019). 

The prevalence of *H. pylori* infection varies according to the geographic region (de Martel et al., 2018). Countries with the highest cancer incidence also have high *H. pylori* infection rates (Ferlay et al., 2015; Pereira-Marques et al., 2019). One of such countries is Kazakhstan - a multiethnic state with a diverse cultural heritage (Bray et al., 2018).

*H. pylori* infection is also associated with several risk factors - demographic, socioeconomic status, environmental and sanitation factors, as well as diet and lifestyle habits (Ozbey and Hanafiah, 2017; Travis et al., 2010). A previous Kazakhs study, revealed higher *H. pylori* positivity among persons with low educational and occupational levels, in persons who grew up in the villages and were river water drinkers, as well as among those who used outdoor facilities (Nurgalieva et al., 2002).

Previously our group published data from the same cohort suggesting that the prevalence of gastric mucosal atrophy among asymptomatic individuals in Kazakhstan was very low (Mezmale et al., 2019). However, the gastric cancer incidence was high in this area. The aim of the present study was to analyse the prevalence of *H. pylori* infection and potential risk factors among asymptomatic subjects in the population of Kazakhstan. 

## Materials and Methods

*Study design and participants*

The study was performed as a part of the regional pilot study of GISTAR (Leja et al., 2017) project in collaboration with Kazakh Institute of Oncology and Radiology, Almaty Diagnostic Centre, Semey Regional Oncology Centre. 

The potentially eligible patients were identified among the outpatient clinics of the diagnostic centres in two Kazakhstan cities – Almaty and Semey. Individuals aged 40 to 64 years, who attended the clinic, were invited to participate in the study. The exclusion criteria for participants were: a personal history of gastric or colorectal cancer, gastric resection due to benign disease, self-reported or documented *H. pylori* eradication therapy in the past, antibiotic use within one month prior to the enrolment, proton pump inhibitors or bismuth-containing drug use within two weeks prior enrolment, presence of alarm symptoms for digestive or any other diseases.

A specially trained staff took blood samples, recorded the anthropometric measurements of the study participants and administered the standardized GISTAR (Leja et al., 2017) questionnaire.

*Sample collection and laboratory methods*

A detailed standard operating procedure for biospecimen collection has been developed according to the study protocol. A 10-ml blood sample of each participant was obtained from the antecubital vein in the morning after overnight fasting. After the blood sample was received, it was stored at –80oC until the laboratory analyses were conducted. All samples were collected in regional collaborating centres in Kazakhstan and transported to Riga, Latvia for further analysis.

The antibodies to *H. pylori* were analysed in plasma by latex agglutination method (LZ Eiken *H. pylori* antibody; Eiken Chemical Co. Ltd., Tochigi, Japan). The cut-off for *H. pylori* positivity was ≥10 U/mL.

*Questionnaire data collection, measurement of anthropometric elements*

Each study participant was interviewed by trained staff. The questionnaire captured the information regarding demographic (age, gender, ethnicity), socioeconomic characteristics (marital status, education level, household population, family income, employed status), and personal habits (regular smoking, general alcohol consumption, increased salt intake - always use extra salt with meals, intake of spicy food). 

The core elements of anthropometry such as height, weight and body circumference (waist, hip) of all participants were measured. Further, a body mass index (BMI) was calculated. The BMI was classified into four categories: underweight (<18.5), normal weight (18.5 – 24.9), overweight (25.0 – 29.9), obese (≥30) (NIH, 2020). 

*Statistical analysis*

All statistical analyses were performed using SPSS 20.0 (Statistical Package for Social Sciences, IBM Corporation, USA). The baseline characteristics of study participants with or without *H. pylori* infection were compared, using the Chi-square test. Descriptive analysis was conducted, and continuous variables were reported using mean ± SD; for categorical variables we used frequencies with percentages. As well as the participants were categorized by age into two groups: less or equal to 50 years and older than 51 year. 

Multiple logistic regression analysis was performed to identify independent risk factors for *H. pylori* infection. Odds ratio (OR) and 95% confidence intervals (CI) for the association between *H. pylori* infection and potential risk factors were estimated. Variables were selected for entry into the regression model if potential risk factors were significantly associated with *H. pylori* infection (P-value <0.05) in the univariate analysis, and those variables which were a borderline significant (P-value <0.09). All statistical tests were two-sided and statistical significance level was set at the 5% level.

*Ethical considerations*

The study protocol was reviewed and approved by the Ethics Committee of Riga East University Hospital Support Foundation - 06.10.2016., reg. No.10/16 in Riga, Latvia. Signed informed consent was obtained from all participants before enrollment in the study.

## Results

Altogether 166 individuals from the general population in Kazakhstan were included from three study centres: from Kazakh Institute of Oncology and Radiology – eight (5%) individuals; from Almaty Diagnostic Centre - 32 (19%); from Semey Regional Oncology Centre - 126 (76%) individuals.

*Demographic data and anthropometric measurements*

Out of 166 participants, 98 (59%) were male and 68 (41%) female; the median age of study subjects was 51 (range from 40 to 64 years). There was no statistically significant difference between gender in respect to the average age (P=0.38). 

The majority of the study subjects - 112 (67.5%) were Kazakhs, 46 (27.7%) were Russians and 8 (4.8%) were representatives of other ethnic groups including Chechen, Kurdish, Tatar, Uyghur, Korean, Azerbaijanis. 

*H. pylori* seropositivity did not differ between men and women: 65 (65.5%) vs 39 (57.5%) (P=0.24). From all Kazakhs 74 (66.1%) were *H. pylori* positive and this ethnic group had a higher proportion of *H. pylori* infection than other ethnic groups (P=0.05). Age group >50 years was also associated with the presence of infection. The demographic features of 166 participants with and without *H. pylori* infection are summarized in Table 1.

Anthropometric measurements of the two study groups are summarized in Table 2. There were no statistically significant differences between *H. pylori* positive and negative groups based on anthropometric measurements (P>0.05).

*Socioeconomic characteristics and personal habits*

The socioeconomic features and personal habits of study subjects in relation to the presence of *H. pylori* infection are summarised in Table 3. No significant difference was observed between socioeconomic factors and *H. pylori* prevalence (P > 0.05). However, participants who reported increased salt consumption were more likely to be *H. pylori* positive (P=0.04). 

*Prediction of H. pylori infection risk*

The final multiple logistic regression model included the following variables - increased salt intake, age groups and nationality (Table 4). Only increased salt consumption and age group older than 50 years were significantly associated with infection. 

**Table 1:** Demographic Features of Participants with and without *H. pylori* Infection, Univariate Analysis

*H. pylori* infection		Total positive no.	%	Total negative no.	%	P-value
Gender	Male	65	66.3	33	33.7	0.24
	Female	39	57.4	29	42.6	
Age (years)	≤50	23	30.3	53	69.7	0.08
	>50	39	43.3	51	56.7	
Ethnicity	Kazakhs	74	66.1	38	33.9	0.05
	Russians	23	50	23	50	
	Other	7	87.5	1	12.5	

no, number

**Table 2:** Anthropometric Measurements of Participants with And without *H. Pylori *Infection, Univariate Analysis

*H. pylori* infection	Total positive no.	%	Total negative no.	%	P-value
BMI (kg/m^2^)					
<18.5	1	100	0	0	
18.5 – 24.9	30	57.7	22	42.3	0.64
25.0 – 29.9	44	67.7	21	32.3	
>30	29	60.4	19	39.6	
	Positive, mean ± SD		Negative, mean ± SD		
Waist circumference (cm)	96.2 ± 13.7		93.2 ± 15.4		0.18
Hip circumference (cm)	106.2 ± 10.0		106.8 ± 12.9		0.81

BMI, body mass index; SD, standard deviation; cm, centimeters, no., number

**Table 3:** Relationships between The Prevalence of *H. pylori *Infection and Potential Risk Factors, Univariate Analysis

*H. pylori* infection		Total positive no.	%	Total negative no.	%	P-value
Living in a household	Alone	3	42.9	4	57.1	0.26
	With family members	100	63.7	57	36.3	
Family status	Married	85	63	50	37	0.96
	Single	19	63.3	11	36.7	
Number of children	≤2	76	61.8	47	38.2	0.69
	>2	28	65.1	15	34.9	
Siblings	≤2	45	63.4	26	36.6	0.86
	>2	59	62.1	36	37.9	
Education level	Basic	70	63.1	41	36.9	0.87
	Higher	34	61.8	21	38.2	
Employed status	Unemployed	79	66.4	40	33.6	0.11
	Employed	25	53.2	22	46.8	
Family income per month	≤100 €	65	60.2	43	39.8	0.33
	>100 €	38	67.9	18	32.1	
Alcohol drinking	Yes	50	61	32	39	0.65
	No	54	64.3	30	35.7	
Cigarette smoking	Yes	67	66.3	34	33.7	0.22
	No	37	56.9	28	43.1	
Intake of spicy food	Yes	33	66	17	34	0.55
	No	71	61.2	45	38.8	
Increased salt intake	Yes	54	56.3	42	43.7	0.04
	No	50	71.4	20	28.6	

no, number

**Table 4:** Multiple Logistic Regression Model for Predicting the Risk for *H. pylori* Infection

*H. pylori *infection		Adjusted OR	95% CI	P-value
Increased salt intake	Yes	2.21	1.12 – 4.35	0.02
	No	1	-	-
Age group	>50	1.99	1.03 – 3.86	0.04
	≤50	1	-	-
Nationality	Other	0.28	0.03 – 2.44	0.24
	Russian	1.64	0.79 – 3.40	0.18
	Kazakhs	1	-	-

OR, odds ratio, CI, confidence interval.

## Discussion

To our knowledge, this is the most up-to-date information on prevalence and potential risk factors for *H. pylori* infection among asymptomatic individuals in Kazakhstan. 

Since the incidence of gastric cancer in Kazakhstan is high and ranks at the third-place from all cancers (Globocan, 2018), a high prevalence of *H. pylori* infection was expected. The idea was supported by our results showing that the overall prevalence of *H. pylori* infection in the study population was 62.7%. However, previous studies in Kazakhstan have demonstrated even higher infection rate: the overall prevalence of *H. pylori* seropositivity in 2010 among asymptomatic adults was 86% and 64% among children (Benberin et al., 2013), while in symptomatic population *H. pylori* infection was found to be 62% in 2002 (Nurgalieva et al., 2002). Nevertheless, the prevalence of infection in Kazakhstan in our study was higher than in the neighbouring countries: the latest data show that the prevalence of *H. pylori* infection in Kyrgyzstan was 51,9% (Dzhumabaev et al., 2015), in China – 52,2% (W. Wang et al., 2019), and in Russia – 53% (Plavnik RG, 2019). Higher infection rate has been reported only in Uzbekistan, where the prevalence of *H. pylori* infection was 74.9% (Abdiev et al., 2010). It should be noted, that both countries – Kazahstan and Uzbekistan showed a high incidence of gastric cancer in 2018: in Uzbekistan incidence rate of gastric cancer was 10,4 per 100 000 population, meanwhile, in Kazakhstan - 15,7 per 100 000 inhabitants (Globocan, 2018). 

Increased age is generally accepted as a risk factor for *H. pylori* infection and many studies report increasing prevalence of *H. pylori* infection with increasing age (S. Chen et al., 2013; Hirayama et al., 2014; Syam et al., 2015; W. Wang et al., 2019). Similarly, our team found that *H. pylori* infection was more likely in an age group older than 50, and the age was a significant predictor for *H. pylori* infection. In the previous study conducted in 2002 by Kazakhstan scientists have reported that the *H. pylori* prevalence increases from 40 years onwards and the highest prevalence was found in the age group of 50-59 (Nurgalieva et al., 2002).

Although univariate analysis showed a significantly higher prevalence of *H. pylori* among Kazakhs compared to Russians, in the multivariate analysis ethnicity was not found to be a significant predictor for *H. pylori* infection. Similarly, Nurgalieva and team did not find any association between *H. pylori* infection Kazakhs and Russians (Nurgalieva et al., 2002). 

Possible factors that could interact with ethnicity in the statistical analysis could be dietary habits. In our study, increased salt intake was a significant risk factor for *H. pylori* infection in the multivariate analysis. We have to note that 57,8% of study participants reported use of extra salt with meals. According to the World Health Organization (WHO) data, salt intake in Kazakhstan stands at about 17 grams per day, which is more than three times above the WHO recommended daily intake (WHO, 2019). It could be related to traditional cooking - people use salt not only for meat and sausages, but also for traditional yoghurt products such as kurt and airan (WHO, 2019). *H. pylori* infection together with increased salt consumption may play an important role in gastric cancer development. For example, salty food could cause temporary damage of the gastric mucosa leading to changes in the viscosity of the gastric mucosal barrier in that way facilitating *H. pylori* colonization and increased risk of developing gastric cancer (Nozaki et al., 2003). Similarly, a study from China demonstrated that patients with increased salt consumption and *H. pylori* have a higher risk of developing gastric cancer (Zhong et al., 2012). A large review of epidemiological studies performed by Wang et al. confirmed a positive association between increased intake of salt and the risk of gastric cancer (Wang et al., 2009). Possibly, high salt diet together with *H. pylori* might explain the high incidence of gastric cancer in Kazakhstan.

Although several studies show an association of *H. pylori* infection with socioeconomic factors, smoking and alcohol consumption (Chen et al., 2014; Wang et al., 2019), none of the studied factors (marital status, education level, employment, smoking and alcohol consumption) in our population sample was related to *H. pylori* infection. The same results were reported by Ogihara et al. (Ogihara et al., 2000) and Shinchi et al. (Shinchi et al., 1997) suggesting that these factors are not independent predictors for *H. pylori* infection. Similarly, no significant association was found between *H. pylori* infection and anthropometric measurements (height, weight and body circumference) although several previous studies show that patients with overweight or obesity are more likely to have *H. pylori* infection (Al-Zubaidi et al., 2018; Lender et al., 2014; Xu et al., 2014).

It should be noted that the study has limitations. The main limitation was the small study population size, thus reducing the statistical power of the study. Despite the proportional distribution between ethnic groups, it would be worthwhile to increase the study population size, to make the results more reliable.

In conclusion, the results of the study demonstrate that the overall prevalence of *H. pylori* infection among asymptomatic middle-aged subjects in Kazakhstan was 62.7% and *H. pylori* infection is independently associated with older age and increased salt consumption. However, factors such as anthropometric measurements, socioeconomic factors were not related to *H. pylori* infection in the studied population. However, it should be noted that we were not able to fully assess this population due to the aforementioned limitations. 

Knowledge of the risk factors of *H. pylori* infection is important because it could prevent the spread of the bacterium in a high-risk population like Kazakhstan, which has a high burden of gastric cancer. 
